# Alisol B 23-acetate activates ABCG5/G8 in the jejunum via the LXRα/ACAT2 pathway to relieve atherosclerosis in ovariectomized ApoE^-/-^ mice

**DOI:** 10.18632/aging.104185

**Published:** 2020-11-25

**Authors:** Xi-Chao Yu, Yu Fu, Yun-Hui Bi, Wei-Wei Zhang, Jun Li, Tingting Ji, Ying Chao, Qing-Hai Meng, Qi Chen, Meng-Hua Ma, Yu-Han Zhang, Jinjun Shan, Hui-Min Bian

**Affiliations:** 1 School of Pharmacy, Nanjing University of Chinese Medicine, Nanjing 210023, China; 2 National Standard Laboratory of Pharmacology of Chinese Materia Medica, Nanjing University of Chinese Medicine, Nanjing 210023, China; 3 Jiangsu Key Laboratory for Pharmacology and Safety Evaluation of Chinese Materia Medica, Nanjing University of Chinese Medicine, Nanjing 210023, China; 4 Jiangsu Key Laboratory of Therapeutic Material of Chinese Medicine, Nanjing University of Chinese Medicine, Nanjing 210023, China; 5 Institute of Pediatrics, Jiangsu Key Laboratory of Pediatric Respiratory Disease, Nanjing University of Chinese Medicine, Nanjing 210023, China; 6 Medical Metabolomics Center, Nanjing University of Chinese Medicine, Nanjing 210023, China

**Keywords:** AB23A, ABCG5/G8, LXRα-ACAT2, lipid mass spectrometry, exogenous cholesterol

## Abstract

Phytosterols have been shown to improve blood lipid levels and treat atherosclerosis. This research investigated the effects of phytosterol Alisol B 23-acetate (AB23A) on jejunum lipid metabolism and atherosclerosis. The results show that intragastric administration of AB23A can significantly reduce atherosclerotic plaque area and lipid accumulation in the jejunum of ovariectomized ApoE^-/-^ mice fed a high-fat diet and can also improve the lipid mass spectra of the plasma and jejunum. In vitro studies have shown that AB23A can increase cholesterol outflow in Caco-2 cells exposed to high fat concentrations and increase the expression of ATP-binding cassette transfer proteins G5/G8 (ABCG5/G8), the liver X receptor α (LXRα). Furthermore, inhibition of LXRα can significantly eliminate the active effect of AB23A on decreasing intracellular lipid accumulation. We also confirmed that AB23A has a negative effect on Acyl-CoA cholesterol acyltransferase 2 (ACAT2) in Caco-2 cells cultured in the high concentrations of fat, and we found that AB23A further reduces ACAT2 expression in cells treated with the ACAT2 inhibitor pyripyropene or transfected with ACAT2 siRNA. In conclusion, we confirmed that AB23A can reduce the absorption of dietary lipids in the jejunum by affecting the LXRα-ACAT2-ABCG5/G8 pathway and ultimately exert an anti-atherosclerotic effect.

## INTRODUCTION

Menopause is caused by the depletion of the ovarian hormones estrogen and progesterone due to a reduction in the limited storage of the ovarian follicles [1, 2]. Menopause usually begins in women in their mid-to-late 40s. The complex physiological processes associated with menopause are usually accompanied by other effects of aging and social adaptation [3]. The onset of menopause is accompanied by unfavorable levels of cardiovascular risk factors, metabolic diseases, osteoporosis, sexual dysfunction and premature cognitive decline. [4]. Decreased ovarian function in postmenopausal women can lead to changes in lipid metabolism and increase the risk of cardiovascular and cerebrovascular diseases, especially the risk of myocardial infarction and angina caused by atherosclerosis, the timing of the increased incidences of these diseases is consistent with menopause [5, 6]. J Witteman J, who surveyed 294 premenopausal women and 319 postmenopausal women with atherosclerosis between the ages of 45 and 55 by radiography, concluded that postmenopausal women are 3.4 times more likely to develop atherosclerosis than premenopausal women (95% confidence interval is 1.2 to 9.7; p <0.05) [7]. The results of animal experiments performed by Meng Q showed that ovarian removal can exacerbate the abnormal levels of blood lipids and pathological changes of aortic roots in ApoE^-/-^ mice [8]. Yang Q [9] also proved that removing ovaries can exacerbate the development of atherosclerosis in LDL^-/-^ mice. Studies on atherosclerosis (AS) have confirmed that it is a chronic metabolic disorder which is closely related to the inflammatory response and dyslipidemia [10]. Rose R [11] stated in the theory of injury response, that lipids and their modified products in the blood can induce the formation of atherosclerotic plaques, which eventually leads to the failure of tissues and organs supplied by the arteries, such as heart failure [12] and brain atrophy [13]. In addition, the lipids in the cores of necrotic plaques, increase the plaque instability and cause acute myocardial infarction and stroke [14]. Therefore, adjusting blood lipid levels is an important method for preventing and treating atherosclerosis in postmenopausal women and reducing the mortality of cardiovascular events in postmenopausal women.

The mucosal layer of the proximal jejunum is a key site of exogenous lipid metabolism [15]. Intestinal epithelial cells on the villi of the small intestine express various functional proteins that are involved in absorption, efflux, synthesis and transport. For example, the absorption of cholesterol in the small intestine directly depends on the NPC1L1 protein on the surface of intestinal epithelial cells, and ezetimibe reduces the absorption of exogenous lipids by inhibiting NPC1L1 in the small intestine [16]. A portion of the free cholesterol absorbed into the cell is esterified to cholesterol ester by acyl-CoA-cholesterol acetyltransferase 2 (ACAT2) in the endoplasmic reticulum [17]. Most of the free cholesterol that is not esterified by ACAT2 is transported back to the intestinal cavity through ABCG5 and ABCG8 [18], the other portion is involved in the synthesis of preβ1-HDL derived from the small intestine; this portion is transported from the basal side of the intestinal epithelial cells to the capillaries in the center of intestinal villi via ABCA1 and enters the blood circulation through the portal vein [19]. Intracellular triglycerides are also recruited into the endoplasmic reticulum through microsomal triglyceride transfer protein (MTP) [20]. Finally, with the participation of class B scavenger receptor 1 (SR-B1) [21], cholesterol is assembled into chylomicrons (CM) with apolipoproteins B48, C, AI, AIV, phospholipids and cholesterol esters [22] and secreted into capillary lymphatic vessels [23]. Then, increased ABCG5/G8 expression in small intestinal epithelial cells can directly reduce exogenous cholesterol in the blood and indirectly regulate triglyceride and phospholipid metabolism.

Several studies have confirmed that phytosterols have anti-inflammatory, liver-protecting, and lipid-lowering functions [24]. Alissonide B 23-acetate (AB23A) is a derivative of the plant sterol Alisinol B, which is extracted and isolated from the medicinal plant Alisson. The partial results of its pharmacological studies on AB23A are as follows: AB23A can reduce the levels of IL-6, IL-1β and TNF-α in the plasma of mice with cardiac dysfunction induced by LPS, reduce myocardial inflammation and infiltration, and improve the survival rate of model mice [25]. AB23A can prevent nonalcoholic steatohepatitis and cholestatic liver injury in mice by activating FXR [26, 27]. However, the mode and mechanism of action of AB23A in regulating lipid metabolism have not been fully elucidated. To further explore the pharmacological effects of AB23A and the underlying mechanisms, we hypothesized that AB23A can also regulate blood lipids in a manner that interferes with lipid metabolism in the small intestine. In this research, we screened plasma lipids and small intestine lipids that were significantly regulated by AB23A through nontargeted lipidomics, and studied targets for lipid metabolism in the small intestine. We also demonstrated the mechanism by which AB23A affects lipid metabolism in the small intestine by treating intestinal Caco-2 cells with high levels of cholesterol.

## RESULTS

### Supplementation with AB23A can reduce atheromatous plaque formation and improve lipid profiles in ovariectomized ApoE^-/-^ mice fed a high-fat diet

**Figure 1:**
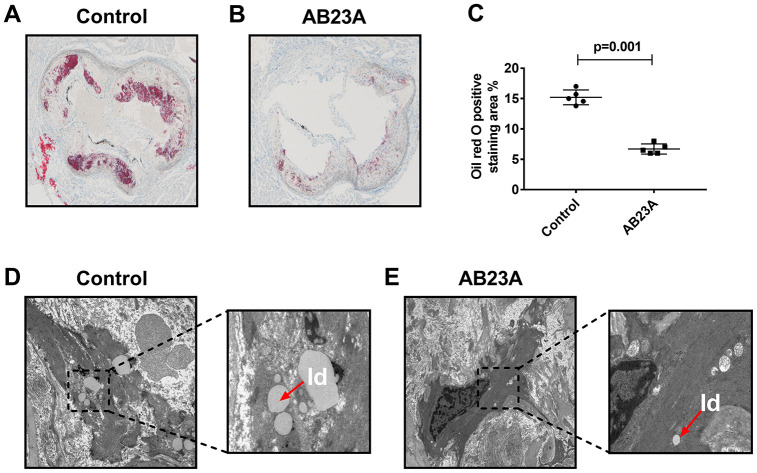
**AB23A improves atherosclerotic lesions in ovariectomized ApoE^-/-^ mice.** Ovaries were removed from 8-week-old female ApoE^-/-^ mice, and the mice were fed a high-fat diet with saline or AB23A (2.55 mg/kg) daily for 12 weeks. (**A**, **B**) Representative image of oil red O staining of the aortic arch. Original magnification: 40×. (**C**) Quantification of lipid area in plaques (n=5/group) The P-value represents the comparison with the control group. The data are expressed as the mean ± SEM (n=5/group). (**D**, **E**) TEM image of the aortic arch of ovariectomized ApoE^-/-^mice. Red arrow, lipid droplets. The original magnifications of the images in the same group are 1.2k× (left) and 5.0k× (right).

**Figure 2:**
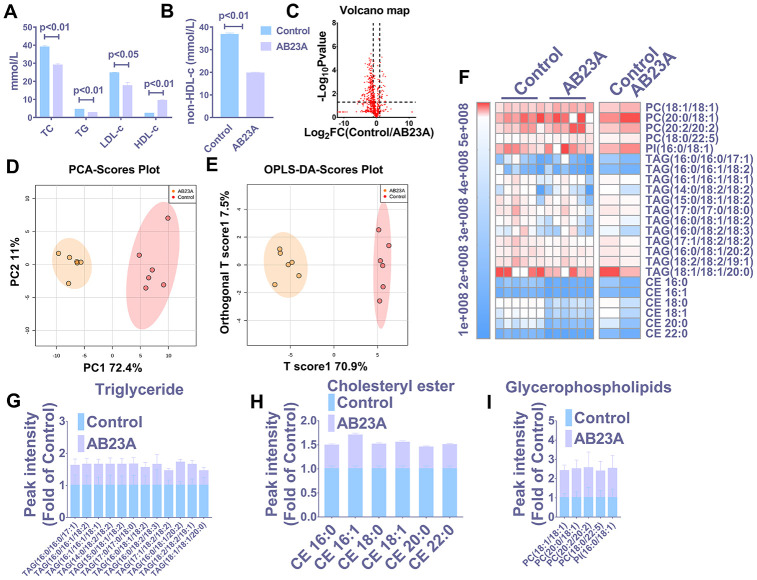
**AB23A improves the plasma lipid fraction of ovariectomized ApoE^-/-^ mice.** (**A**, **B**) Kits were used to measure the levels of total cholesterol (TC), total triglycerides (TG), high-density lipoprotein (HDL-c) and low-density lipoprotein in plasma (LDL-c). The remaining plasma lipids were extracted and detected by HPLC-Q-TOF/MS, and statistical analysis was performed according to different mathematical models. (**C**) The volcano plot shows the difference in the lipid metabolites between the AB23A-treated group and the control group. (**D**) PCA score chart showing the differences in the plasma samples from the AB23A-treated group (green) and the model control group (red). PC1=72.4%, PC2=11.0%. (**E**) The OPLS-DA score chart shows the AB23A-treated group (Orange) and the control group (Red). The adaptability and predictive ability are expressed as follows: T score1=70.9%, Orthogonal T score1=7.5%. (**F**) The heat map shows that after screening, AB23A can significantly affect the different classes of plasma lipids. (**G**–**I**) The box plots show the relative lipid content of each category. The data are expressed as the mean ± SEM (n=6/group).

HE staining results showed that postmenopausal women had significantly more subendothelial lipid vacuoles in the ascending aorta than premenopausal women (Supplementary Figure 1A, 1B). To elucidate the effect of AB23A on the prevention and treatment of atherosclerosis in postmenopausal women, high-fat diet-fed ovariectomized ApoE^-/-^ mice were given AB23A by gavage for 12 weeks. By comparing the body weight of the two groups of mice each week, we found that the body weight of the AB23A group was significantly lower than that of control group (Supplementary Figure 4A). The results of oil red O staining showed that AB23A can significantly reduce the lipid accumulation in the atherosclerotic plate (Figure 1A, 1B) and the lesion area (Figure 1C, oil red data). We observed this phenomenon through TME; compared with the control group, the AB23A group exhibited a reduced volume of lipid droplets in the aortic roots (Figure 1D, 1E). As shown in Figure 2A, 2B, Lipids (TC TG HDL LDL), compared with the control group, the AB23A-treated group had significantly lower plasma TC, TG, non-HDL-c and LDL-c levels and significantly higher plasma HDL-c levels. The remaining mouse plasma was used for nontargeted lipidomics research, and the results showed that under different mathematical models, there were significant differences between the lipid metabolites of the two groups (Figure 2C–2E, Lipidomics). The statistical results showed that AB23A can reduce the levels of 6 cholesterol esters and 12 triglycerides and increase the levels of 5 glycerophospholipids in the plasma (Figure 2F–2I, Lipidomics). These results indicate that AB23A improves the blood lipid levels in the body and inhibits the progression of AS.

### Supplementation with AB23A can improve jejunum morphology and regulate jejunal lipid levels in ovariectomized ApoE^-/-^ mice fed a high-fat diet

**Figure 3:**
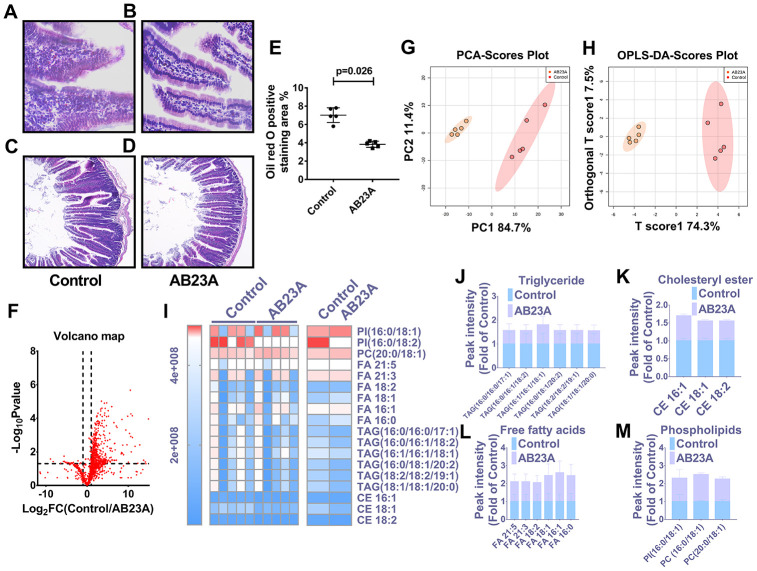
**AB23A maintains the structural stability of the jejunum in ovariectomized ApoE^-/-^ mice by improving the jejunal lipid mass spectrum.** Ovaries were removed from 8-week-old female ApoE^-/-^ mice, and the mice were fed a high-fat diet with saline or AB23A (2.55 mg/kg) daily for 12 weeks. The proximal jejunum lipids were extracted and detected by HPLC-Q-TOF/MS, and statistical analysis was performed according to different mathematical models. (**A**, **B**) Representative image of oil red O staining of the proximal jejunum. Original magnification: 40×. (**C**, **D**) Representative HE staining of ovariectomized ApoE^-/-^ mice with proximal jejunal lesions. Original magnification: 20×. (**E**) Quantification of the lipid area in the villi (n=5/group). p-values represent comparisons with the control group. The data are expressed as the mean ± SEM (n=5/group). (**F**) The volcano plot shows the difference in the lipid metabolites between the AB23A-treated group and the control group. (**G**) The PCA score chart shows the differences in the plasma samples from the AB23A-treated group (Orange) and the model control group (Red). PC1=84.7%, PC2=11.4%. (**H**) The OPLS-DA score chart shows the AB23A-treated group (Orange) and the control group (Red). The adaptability and predictive ability are expressed as follows: T score1=74.3%, Orthogonal T score1=19.7%. (**I**) The heat map shows that after screening, AB23A can significantly affect the different classes of plasma lipids. (**J**–**M**) The box plots show the relative lipid content of each category. The data are expressed as the mean ± SEM (n=5/group).

In light of the close relationship between excessive intake of dietary lipids and atherosclerosis, we further verified whether the protective effect of AB23A on atherosclerosis is related to its role in regulating jejunal lipid metabolism. The results showed that AB23A reduced the lipid accumulation in the jejunum villi (Figure 3A, 3B, 3E, oil red data), and restored the structural stability of the jejunum villi (Figure 3C, 3D). The remaining jejunum villi were used for nontargeted lipidomics research (Figure 3F–3I, Lipidomics). The statistical results showed that AB23A can reduce the levels of 3 cholesterol esters and 6 triglycerides, and increase the levels of 3 glycerophospholipids in the jejunum tissue. It is worth noting that the 2 kinds of cholesterol esters,6 kinds of triglycerides, and 2 kinds of glycerophospholipids that produce significant changes in the jejunum coincide with the significantly changes in the lipids in the plasma. In addition, we found that AB23A significantly increased the content of free fatty acids (FAs) in the jejunum (Figure 3J–3M, Lipidomics). Compared with the main fatty acids in the diet (Table 1), we found that after intervention with AB23A, oleic acid (18:1), palmitic acid (16:0), linoleic acid (18:2) and palmitoleic acid (16:1) were significantly elevated in the jejunum. We found that the relative abundance of cholesterol ester (18:1), cholesterol ester (18:2) and cholesterol ester (16:0) containing the abovementioned carbon chain fatty acyl group were significantly reduced in the jejunum tissue of the AB23A group. These results revealed that AB23A can maintain the structural stability of the mouse jejunum under high-fat diet feeding, and participate in regulating lipid metabolism in the jejunum. In addition, AB23A affects blood lipid levels by regulating jejunum lipid metabolism. We speculated that AB23A interferes with the metabolism of free cholesterol in the jejunum, but its specific mechanism of action remains to be further explored.

**Table 1:** The main fatty acids in the diet.

**Customary name**	**Number of carbon atoms and double bonds**	**Molecular formula**
oleid acid	18:1	CH^3^(CH^2^)^7^CH=CH(CH^2^)^7^COOH
palmitic acid	16:0	CH^3^(CH^2^)^14^COOH
stearic acid	18:0	CH^3^(CH^2^)^16^COOH
linoleic acid	18:2	CH^3^(CH2)^4^(CH=CHCH^2^)^2^(CH^2^)^6^COOH
palmitoleic acid	16:1	CH^3^(CH^2^)^5^CH=CH(CH^2^)^7^COOH
myristic acid	14:0	CH^3^(CH^2^)^12^COOH
α-linolenic acid	18:3	CH^3^CH^2^(CH=CHCH^2^)^3^(CH^2^)^6^COOH
arachidonic acid	20:4	CH^3^(CH^2^)^4^(CH=CHCH^2^)^4^(CH^2^)^4^COOH

### Supplementation with AB23A can affect targets which related to exogenous lipid metabolism in the jejunum of ovariectomized ApoE^-/-^ mice fed a high-fat diet

**Figure 4:**
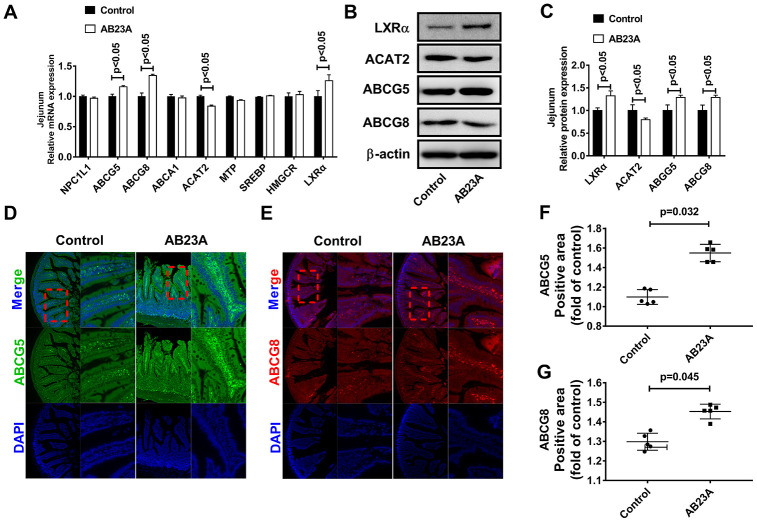
**AB23A reduces the ability of the proximal jejunum to metabolize exogenous lipids into the blood.** (**A**) mRNA expression of targets related to lipid metabolism in the jejunum. (**B**, **C**) Western blot was used to detect the protein levels of LXRα, ABCG5, ABCG8 and ACAT2 in the proximal jejunum. (**D**–**G**) Immunofluorescence detection of ABCG5 and ABCG8 in the proximal jejunum. p-values represent comparisons with the control group. The results represent the mean ± SEM (n=3/group).

Exogenous lipids need to undergo multiple reaction steps and are metabolized into the blood by the jejunum in different ways. Furthermore, the jejunum tissue also has the ability to synthesize lipids de novo. Next, we explored the effects of AB23A on jejunal lipid metabolism-related targets. We analyzed the expression of lipid metabolism-related targets in the jejunum tissues of different groups by qRT-PCR, Western blot and immunofluorescence. The results showed that AB23A significantly increased LXRα, ABCG5/G8, and ACAT2 mRNA in the jejunum tissues, but had no significant effect on NPC1L1, SR-B1, MTP, LXRβ, SREBP1, HMGCR, ACC, and ACS (Figure 4A, WB and RT-PCR data, IF data). The results of WB and IF were consistent with those of qRT-PCR (Figure 4B–4G, WB and RT-PCR data). From the results described above, we can draw the conclusions that AB23A mainly affects the many targets for exogenous lipid metabolism in the jejunum, reduces the metabolism of lipids into the blood in the jejunum tissue, and plays a role in regulating blood lipids and preventing atherosclerosis.

### AB23A can reduce intracellular lipid accumulation in Caco-2 cells under high-fat culture conditions

**Figure 5:**
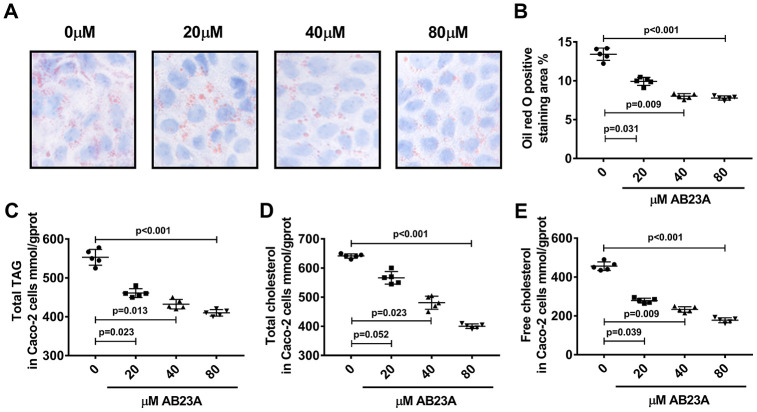
**AB23A reduces lipid accumulation in Caco-2 cells cultured under high-fat conditions. Caco-2 cells cultured under high-fat conditions were exposed to different concentrations of AB23A (0, 20, 40, or 80 μM) for 24 h.** (**A**) Representative image of Oil Red O staining of Caco-2 cells. Original magnification: 200×. (**B**) Quantification of the intracellular lipid droplet area (n=5/group). (**C**–**E**) Kits were used to measure the levels of total cholesterol, total triglycerides, free cholesterol, and cholesterol esters in Caco-2 cells. All the results were obtained from three independent experiments. p-values represent comparisons with controls. The values represent the mean ± SEM (n=6/group).

Next, we investigated the effects of AB23A on the lipid metabolism of Caco-2 cells under high-fat culture conditions via in vitro experiments. The Caco-2 cells were allowed to adhere to the bottom of petri dishes, and we waited for the cells to form polygonal tight connections. The cell culture conditions were changed to high-fat conditions, and the cells were exposed to different concentrations of AB23A (0, 20, 40, or 80 μM) and incubated for 24 h. Oil red O staining, a total triglyceride detection kit, a total cholesterol detection kit, a cholesterol ester detection kit, and a free cholesterol detection kit were used to evaluate the accumulation of intracellular lipid droplets and the content of cholesterol and triglycerides. The MTT results showed that the AB23A concentration used above was not cytotoxic to the Caco-2 cells (Supplementary Figure 2A, 2B, Biochemical Indicators). The results of the in vitro experiments were consistent with the results of the in vivo studies. Compared with the control group, the AB23A-treated group exhibited reduced intracellular accumulation of lipid droplets in a dose-dependent manner (Figure 5A, 5B, oil red data). The results of the rapid test kit showed that the contents of triglyceride (TG), total cholesterol (TC), cholesterol ester (CE) and free cholesterol (FC) in the cells decreased in a dose-dependent manner (Figure 5C–5E, Lipids(TC TG HDL LDL)). In summary, AB23A can reduce the lipid content of intestinal Caco-2 cells under high-fat culture conditions.

### AB23A can induce the expression of ABCG5/G8 in Caco-2 cells under high-fat culture conditions

**Figure 6:**
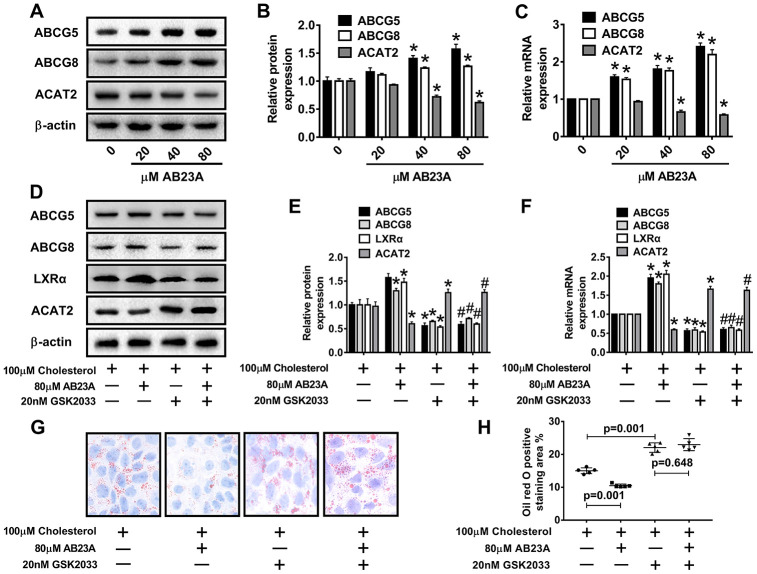
**AB23A promotes the expression of ABCG5 and ABCG8 by activating LXRα.** Caco-2 cells cultured under high-fat conditions were exposed to different concentrations of AB23A (0, 20, 40, or 80 μM) for 24 h. (**A**–**C**) The protein and mRNA expression of ACAT2, ABCG5 and ABCG8 in Caco-2 cells from different groups (n=3/group). (**D**–**F**) The LXRα inhibitor GSK2033 was added with 80 μM AB23A for 24 h. RT-qPCR and Western blot analyses were performed to evaluate the mRNA and protein levels of LXRα, ACAT2, ABCG5, and ABCG8 (n=3/group). (**G**) Representative image of Oil Red O staining of Caco-2 cells. Original magnification: 200×. (**H**) Quantification of the intracellular lipid droplet area (n=5/group). The data are expressed as the mean ± SEM, and the results were obtained from three independent experiments. *P <0.05 compared to the control group; #P <0.05 vs the AB23A only group.

ABCG5 and ABCG8 are two pivotal proteins in small intestinal epithelial mucosal cells that mediate the release of intracellular free cholesterol from the extracellular space. To determine the potential mechanism by which AB23A reduces intracellular lipid accumulation in Caco-2 cells, we treated Caco-2 cells under high fat culture conditions with different concentrations of AB23A (0, 20, 40, or 80 μM) for 24 h, and then, Western blot and qRT-PCR analyses were performed. The results show that AB23A could effectively enhance the protein and mRNA expression of ABCG5 and ABCG8 in Caco-2 cells, and these effects were concentration-dependent. We also found that AB23A could significantly inhibit the protein and mRNA expression of ACAT2 in Caco-2 cells cultured under high-fat conditions and positively regulate the protein and mRNA expression of ABCG5/G8. (Figure 6A–6C, WB and RT-PCR data). In addition, AB23A treatment increased the protein expression of ABCA1 and NPC1L1 by a small amount, although these effects were not statistically significant, and we also found that the administration of 80 μM AB23A to Caco-2 cells could significantly inhibit the protein expression of CYP7A1 but had no significant effect on the protein expression of ABCG1 (Supplementary Figure 2C, 2D, WB and RT-PCR data). Therefore, AB23A can reduce the accumulation of cholesterol in Caco-2 cells in vitro and the accumulation of exogenous cholesterol in the jejunum by increasing the expression of ABCG5/G8.

### Role of AB23A-induced LXRα-ACAT2 pathway activation in ABCG5/G8 expression and cholesterol homeostasis in Caco-2 cells maintained under high-fat culture conditions

**Figure 7:**
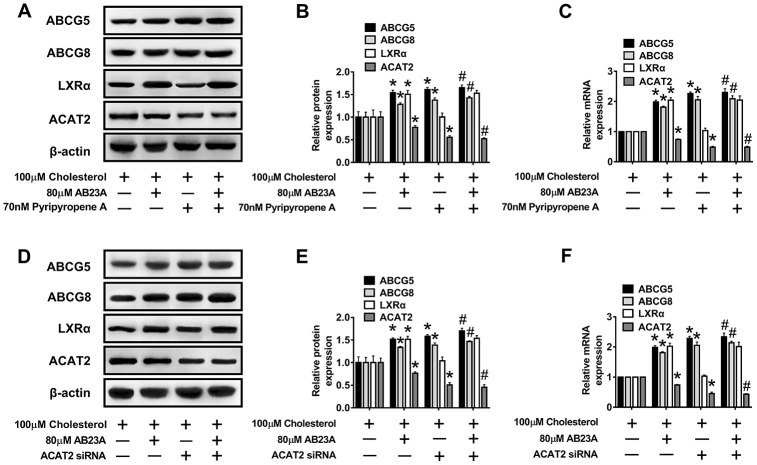
**The LXRα-ACAT2 pathway is involved in the upregulation of ABCG5 and ABCG8 by AB23A.** In Caco-2 cells cultured under high-fat conditions, 80 μM AB23A and ACAT2 inhibitors were simultaneously administered and incubated for 24 h. (**A**–**C**) RT-qPCR and Western blot analyses were performed to assess the mRNA and protein levels of ABCG5, ABCG8, LXRα, and ACAT2 (n=3/group). Caco-2 cells were pretreated with ACAT2 siRNA. (**D**–**F**) RT-qPCR and Western blot analyses were performed to evaluate the mRNA and protein levels of ABCG5, ABCG8, LXRα, and ACAT2 in the different groups (n=3/group). The data are expressed as the mean ± SEM, and the results were obtained from three independent experiments. *P <0.05 compared to the control group; #P <0.05 vs the AB23A only group.

Numerous studies have confirmed the regulatory effect of LXRα on ATP-binding cassette transporters. First, we examined whether the ability of AB23A to increase ABCG5/G8 in order to improve lipid accumulation depends on LXRα. As shown in Figure 6D–6F, WB and RT-PCR data, the LXRα inhibitor GSK2033 eliminated the positive effects of AB23A on the protein and mRNA expression of ABCG5/G8 and significantly increased the protein and mRNA expression of ACAT2. The results obtained with oil red staining also prove this point (Figure 6G, 6H, oil red data). The research described above showed that LXRα plays a central role in the regulation of ABCG5/G8 expression by AB23A. However, it is not clear whether ACAT2 participates in the regulation of ABCG5/8. Next, we sought to further confirm whether ACAT2 is involved in the regulation of AB23A. Similar to the effect of AB23A, the use of the ACAT2-specific inhibitor PPPA significantly reduced the positive effects on the protein and mRNA expression of ABCG5/G8, but PPPA had no significant effect on the protein and mRNA expression of LXRα (Figure 7A–7C, WB and RT-PCR data). Subsequently, the results described above were confirmed in Caco-2 cells transfected with ACAT2 siRNA (Figure 7D–7F, WB and RT-PCR data). The immunofluorescence results showed that the inhibition of ACAT2 significantly reduced the positive effects of AB23A on the intracellular protein expression of ABCG5/G8 (Supplementary Figure 3A–3D, WB and RT-PCR data). These results indicate that AB23A mainly acts on the LXRα-ACAT2 pathway and ultimately increases the expression of ABCG5/G8. In this way, cholesterol that is not esterified in the cell can be easily transported out of the cell.

## DISCUSSION

Small intestine-derived lipids are an integral part of the plasma lipid profile [28]. Clinically, the administration of drugs that interfere with the process of lipid absorption by small intestinal epithelial cells affects the metabolism of exogenous lipids into the blood, effectively improving the abnormal blood lipid status, and ultimately preventing and treating atherosclerosis cardiovascular and cerebrovascular diseases [29]. In recent years, phytosterols have been shown to have a role in lowering plasma cholesterol [30]. In the current study, we first investigated the effect of AB23A on atherosclerotic plaque formation and jejunum morphology in female ovariectomized ApoE^-/-^ mice fed a high-fat diet. Next, we investigated the effect of AB23A on plasma and jejunum lipid mass spectra through nontargeted lipidomics. Finally, we explored the role of AB23A in the ABCG5/G8-mediated maintenance of cholesterol homeostasis in small intestinal epithelial cells and the underlying mechanisms of this regulation.

In the current research, the administration of AB23A can significantly improved the plasma lipid profiles of female ovariectomized ApoE^-/-^ mice fed by high-fat diet. In addition, AB23A reduced the accumulation of lipids in the aortic root plaques. Furthermore, AB23A significantly reduced the lipid accumulation in the proximal jejunum villi of female ovariectomized ApoE^-/-^ mice fed a high-fat diet, and improved the jejunum lipid mass spectrum. After comparing the plasma lipid mass spectra with the intestinal lipid mass spectra, we identified lipids that can be simultaneously regulated by AB23A. Thus, it was proven that the regulatory effect of AB23A on blood lipids depended, in part, on the effect of AB23A on jejunal lipid metabolism into the blood.

It is well known that proteins related to lipid absorption, such as NPC1L1, and proteins related to lipid efflux, such as ATP-binding cassette transporters, are downstream target genes of LXRα and can directly participate in the regulation of intracellular cholesterol homeostasis. However, our results excluded the possibility that AB23A can directly inhibit the effect of NPC1L1. Considering the upregulation of FXR expression by AB23A, we verified that the upregulation of LXRα, ACAT2 and ABCG5/8 by AB23A was independent of the upregulation of FXR by FXR siRNA (Supplementary Figure 2E, 2F, WB and RT-PCR data). Our results confirmed that AB23A depends on the activation of LXRα to maintain the cholesterol balance of small intestinal epithelial cells. By promoting the reverse transport of free cholesterol in intestinal epithelial cells back to the intestinal lumen, AB23A can reduce the entry of foreign lipids into the blood through the jejunum and ultimately improve the blood lipid levels. In addition, incubating AB23A with high-fat cultured Caco-2 cells can significantly increase ABCG5/G8 expression in cells and reduce intracellular lipid accumulation. Finally, we confirmed that the effect of AB23A on ABCG5/G8 expression and its regulation of jejunal lipid metabolism are closely related to the LXRα-ACAT2 pathway.

AB23A is a phytosterol derivative, and modern pharmacological studies have confirmed that AB23A exerts biological activities against cancer [30] and anti-inflammatory activities [25]. The latest evidence now shows that AB23A can upregulate the expression of FXR in the liver and reduce the damage to the liver in pathological conditions such as: nonalcoholic steatohepatitis and cholestatic liver injury [26, 27]. However, whether the effect of AB23A on blood lipids is mediated by the metabolism of exogenous lipids in the jejunum is not clear. Due to the important role of ABCG5/G8 in regulating the reverse transport of cholesterol in the small intestine epithelial cells back to the intestinal lumen, we propose the hypothesis that the role of AB23A in regulating lipid metabolism in the small intestine is mediated by ABCG5/G8. In mice with atherosclerosis, AB23A can improve the small intestinal lipid mass spectrum and increase the protein expression of ABCG5/G8 in the proximal jejunum. Treatment of Caco-2 cells under high-fat conditions with AB23A also significantly increased ABCG5/G8 protein expression and reduced intracellular lipid accumulation. These findings confirm our hypothesis and enrich the way and mechanism by which AB23A regulates of blood lipids.

Liver X receptor α (LXRα) is a class of ligand-activated transcription factors that regulate the expression of intracellular lipid ligands, maintaining the homeostasis of cells and the body [31]. The classic theory is that the activation of LXRα in macrophages can increase the expression of ABCA1/G1, promote the outflow of intracellular cholesterol, accelerate the reverse cholesterol transport (RCT), reduce the formation of foam cells, and reduce the risk of AS [32, 33]. ABCG5 and ABCG8 have been confirmed to be downstream targets of LXRα, and ABCG5/G8 expression can be directly enhanced by activating LXRα [34]. Numerous studies have confirmed that natural products, including phytosterols, taurine, and soy protein (SP), can regulate intestinal LXRα and/or LXRβ [35]. Jogchum Plat [36] used Caco-2 cells to explore the effects of different types of phytosterols on LXR. The results showed that sitosterol, campesterol and algaesterol can significantly activate LXRα. The half-maximum effect concentrations of the aforementioned phytosterols are 42, 43, and 33 nM, respectively. Furthermore, through the structural modification of plant sterols, such as ergosterol and stigmasterol, Maura Marinozzi [37] obtained some highly effective LXRα agonists. Kaneko E [38] found that the ergosterol derivative YT-32 can more effectively activate LXR and selectively upregulate the expression of intestinal ABC transporters in C57BL/6 mice. A report by Yang Z [32] showed that fucoidan treatment can regulate reverse cholesterol transport (RCT) in C57BL/6J mice fed a high-fat diet, and that in the small intestine, fucoidan treatment can reduce Niemann-Pick C1-like 1 (NPC1L1) expression, and improve ABCG5 and ABCG8 expression, thereby reducing cholesterol absorption and increasing cholesterol excretion. González-Granillo treated high-cholesterol diet-fed wild-type mice with edible soy protein (SP) and found that SP can significantly upregulate the expression of ABCG5 and ABCG8 expression in the liver and intestines, relieve liver fat accumulation, and reduce triglycerides, cholesterol and cholesterol contents in lipoproteins, but these effects of SP are not observed in LXRα^-/-^ mice; these data indicate that by activating the LXRα-ABCG5/G8 pathway, it is possible to effectively maintain the relative stability of body lipid metabolism in the body. The results of the in-vivo experiments in our research showed that compared with the model group, the AB23A-treated female ovariectomized ApoE^-/-^ group had significantly increased LXRα protein levels in the proximal jejunum villi. Through in vitro experiments, we observed that the treatment of Caco-2 cells cultured under high-fat conditions with AB23A could increase the protein expression of LXRα in a concentration-dependent manner, and that the administration of LXRα inhibitors eliminated the ABCG5/G8 overexpression induced by AB23A treatment. These results indicate that LXRα mediates the role of AB23A in maintaining jejunum lipid homeostasis and improving atherosclerosis.

Acetyl coenzyme A acetyltransferase 2 (ACAT2) is an enzyme that esterifies free cholesterol into cholesterol esters in the cell, and controls the intracellular cholesterol storage and outflow. As an enzyme in the cell that esterifies free cholesterol into cholesterol esters, ACAT2 controls the storage and efflux of intracellular cholesterol [39] and reduces the lipotoxicity of excess free cholesterol and fatty acids to cells [40]. ACAT2 expressed in hepatocytes and intestinal epithelial cells profoundly affects the synthesis of endogenous lipids in the liver [41] and the metabolism of exogenous lipids in the small intestine [17, 42]. Ohshiro T [43] administered the ACAT2 selective inhibitor pyrrobutene A (PPPA) to ApoE^-/-^ mice by oral administration. That study found that PPPA can significantly reduce the absorption of cholesterol in the intestine and the cholesterol esters synthesized by the liver to improve atherosclerotic lesions in mice. It has been reported that although the upregulation of ABCA1 expression in the small intestine of ACAT2 knockout mice can partially reduce the decrease in the cholesterol absorption in the intestine, the upregulation of the expression of ABCG5 and the mRNA expression of NPC1L1 is suppressed, leading to significant intestinal excretion of cholesterol increased significantly [44]. However, this report did not indicate whether the changes in the expression of ABCG5 and NPC1L1 were mediated by LXRα. In the current study, we confirmed that AB23A can inhibit the expression of ACAT2 in the proximal jejunum of female ovariectomized ApoE^-/-^ mice, and similar results were obtained in Caco-2 cells under high-fat conditions. In addition, the administration of the LXRα inhibitor GSK2033 significantly reduced the upregulation of ABCG5/G8 protein expression by AB23A; By transfecting Caco-2 cells with the ACAT2 specific inhibitor PPPA or ACAT2 siRNA, we found that the expression of LXRα was not significantly affect and that the expression of ABCG5/G8 was increased. If AB23A was administered simultaneously, the expression of ABCG5/G8 was further upregulated, thus proving that the role of AB23A in regulating exogenous lipid metabolism requires the ACAT2-mediated maintenance of cellular lipid homeostasis through the LXRα-ACAT2-ABCG5/G8 pathway.

It is worth noting that whether the effect of AB23A on atherosclerosis involves other pathways or mechanisms still requires further study. Due to the important role of the liver in lipid and bile acid metabolism, we also examined the effect of AB23A on the pathological changes in the liver and the metabolic targets described above. The statistical results in Supplementary Figure 4B–4D show that the liver weights and liver coefficients of the mice in the AB23A group were significantly lower than those of the mice in the control group and that the plasma ALT and AST levels were significantly reduced after the AB23A treatment. TEM images showed that the volume of lipid vesicles in the livers of the AB23A group was significantly reduced compared to that in the livers of the control group (Supplementary Figure 5A, 5B). In addition, we measured the contents of TC and TG in the liver, and the results showed that AB23A significantly reduced the content of TC in the liver but had no significant effect on the content of TG in the liver (Supplementary Figure 4E, Biochemical Indicators). The above results indicated that AB23A can reduce liver steatosis to a certain extent. Next, we confirmed that AB23A can increase the protein expression of LXRα and its downstream ABCG5/8 in the liver, and we also confirmed that AB23A can significantly increase the protein expression of FXR in the liver (Supplementary Figure 5C, 5D, WB and RT-PCR data). To investigate the effect of AB23A on bile acid metabolism, we examined the total cholic acid in the liver and feces of the mice. The results showed that AB23A can significantly reduce the total bile acid (TBA) in the liver and significantly increase the TBA in the feces (Supplementary Figure 4G, 4H, Biochemical Indicators). Our study also found that the expression of the CYP7A1 protein, which is involved in bile acid synthesis in the liver, was significantly downregulated (Supplementary Figure 5C, 5D) and that the content of TC in the feces was obviously increased after AB23A treatment (Supplementary Figure 4F, Biochemical Indicators). Based on the results described above, we can draw the following conclusions: On the one hand, the increase in the direct excretion of liver cholesterol mediated by ABCG5/8 reduces the cholesterol content in the liver. The relative reduction in the substrate leads to a reduction in the total amount of bile acids synthesized by the liver, which relieves liver cholestasis. On the other hand, the reduced level of liver cholesterol also inhibits the production of triglycerides in the liver. Therefore, although the activation of liver LXRα can promote the de novo synthesis of liver lipids to accelerate the reassembly of liver cholesterol into the blood, the ABCG5/8-mediated excretion of cholesterol by the bile ducts partially eliminates the negative effects of LXRα activation on the liver. In summary, AB23A improves the ability of the liver to metabolize lipids under high-fat conditions by relieving fatty liver degeneration and cholestasis and is also an important mechanism by which AB23A regulates blood lipid levels.

## MATERIALS AND METHODS

### Mice and treatments

Eight-week-old female ApoE^-/-^ mice were purchased from Nanjing Qinglongshan Animal Breeding Center. The entire experiment was performed in an SPF animal room. The indoor temperature was controlled at 22-26° C and the humidity was below 50%. 12 h light-dark cycle, free access to drinking water and food. After adaptive feeding for 1 week on the normal diet, both sides of the ovaries were excised and fed on the normal diet for 1 week. 1 week vaginal smear confirmed that the mice had no estrous cycle. The mice that met the requirements were randomly divided into the control group and the AB23A intervention group (n=10/group), both groups were fed HDF (containing 10% fatty oil, 2% cholesterol, 4% whole milk powder and 0.5% sodium cholate) for 12 weeks. According to the data provided by research preliminary research, we chose 2.55 mg/kg AB23A as a therapeutic dose by intragastric administration to mice. At week 15, mice were sacrificed and blood and tissue samples were collected for further evaluation. Animal experiments strictly follow the "Guidelines for the Management and Use of Experimental Animals" (published in May 2016) issued by the National Science and Technology Publications Publishing Committee. The experimental protocol was approved by the Animal Ethics Committee of Nanjing University of Traditional Chinese Medicine. Anesthesia with sodium pentobarbital (45 mg/kg, 0.1 ml/10 g) was performed before surgery to minimize pain. Mice were sacrificed by intraperitoneal injection of 5 times anesthesia volume of sodium pentobarbital (45 mg/kg, 0.5 ml/10 g).

### Human subjects

From May 2019 to November 2019, female ascending aorta samples were collected from 24 subjects who underwent ascending artery replacement surgery. All surgeries and sample collection were conducted at Nanjing First Hospital. The screening criteria were as follows: female, ≥ 30 years old, ascending aortic aneurysm accompanied by aortic atherosclerosis, and the need for ascending aortic replacement surgery. Ascending aorta samples were histologically assessed by an experienced pathologist for diagnosis. Informed consent was obtained from all patients. The patients were divided into groups based on the age and whether in postmenopausal stage, 12 premenopausal female patients aged 40 to 48 years old in one group, and 12 post-menopausal female patients aged 58 to 65 years old in another group.

### Lesion evaluation

The required tissue samples were perfused with PBS and fixed in 4% paraformaldehyde for 12 h. The specimens were then rinsed in running water for 6 h, soaked in double distilled water for 1 h, and placed in 30% sucrose overnight. Taking the mouse aorta as an example, the heart and the aorta were embedded in OCT, placed in a low-temperature microtome, and frozen at -25° C for more than 2 h. An 8 μm section was cut from the aortic sinus and placed on a glass slide. Proximal jejunum sections were performed as above, and oil red O staining was performed every five consecutive sections to assess lipid deposition. Every three consecutive sections were stained with HE, and the degree of lesion was evaluated. Image Pro Plus software was used to quantify the area of aortic and proximal jejunal lesions and the area of lipid deposition in ApoE^-/-^ mice. Data are expressed as relative Area ± SEM.

### Caco-2 cell culture and model evaluation

Caco-2 cells were obtained from Runyan Biotechnology Co., Ltd. (Nanjing, China) and cultured in DMEM high-sugar medium (5% CO_2_, 10% fetal bovine serum (FBS) and 2% penicillin/streptomycin). 37° C. To induce lipid accumulation in Caco-2 cells, we prepared cholesterol micelles by autoclaving and ultrasound at 37° C for 2h. The cells were incubated with cholesterol micelles (100 μM cholesterol, 390 μM oleic acid, 110 μM Monosterin, 5 mM taurocholate) in DMEM high glucose medium containing 5% FBS for 24 h. Oil Red O staining was performed to assess the formation of intracellular lipid accumulation. Briefly, cells were fixed in a 4% paraformaldehyde solution for 10 min, and washed in 60% isopropanol for 15 s. Next, in a darkened condition, use a freshly prepared oil red O working solution to dye at 37° C for 5 min, and then decolorize with 60% isopropanol for 15 s. After washing with PBS, cells were stained with hematoxylin for 5 min, and washed with PBS. Observe positively stained cells (Red) using an optical microscope (Olympus) and acquire images. The final concentration of GSK2033 used to block LXRα was 20 nM, and the final concentration of pyripyropene A (PPPA) used to block ACAT2 was 70 nM. LXR antagonist GSK2033 and ACAT2 antagonist PPPA2 were the products of MedChemExpress (Shanghai, China).

### Kit for detecting lipids and total bile acids (TBA)

Plasma sample pretreatment: Blood was collected from the posterior orbital venous plexus and placed in a centrifuge tube pre-added with 4% sodium citrate anticoagulant, and immediately mixed upside down. The volume ratio of 4% sodium citrate anticoagulant to blood was 1: 9 (V:V), centrifuge at 3000 rpm for 10 min and take the supernatant; Tissue and fecal sample pretreatment: add 100 μl phosphate buffer saline (PBS) per 10mg sample, grind at 4° C, collect suspension, add equal volume of methanol, shake vigorously, centrifuge at 12000rpm, 4° C, for 10min, take the supernatant; Cell sample preparation: add 100 μl PBS per one million cells, grind at 4° C, collect suspension, add equal volume of methanol, shake vigorously, centrifuge at 12000rpm, 4° C, for 10min, take the supernatant. Detect TC, TG, LDL-c, HDL-c and TBA levels according to the instructions of the kit (Nanjing Jiancheng Biotechnology Research Institute, China).

### Methodology for nontargeted lipidomics

Sample preparation: Pipet 20 μL of mouse plasma or proximal jejunum tissue into a centrifuge tube, and add 225 μL of internal standard (Lyso PE (17: 1), SM (17: 0), PE (17: 0/17: 0) Ice methanol with a concentration of about 5 μg/mL), vortex for 10 s, add 750 μL MTBE, vortex for 10 s, shake at 4° C for 10 min, add 188 μL deionized water, vortex for 20 s and centrifuge at 4° C, 18000 rpm for 2 min, Pipette 350 μL of the supernatant into a 1.5 mL centrifuge tube, place it in a centrifugal concentrator and dry it for 2 h. Methanol: toluene was used to dissolve the sample in 110 μL of 9: 1 solution, vortexed for 15 min, sonicated for 15 min, and centrifuged at 18000 rpm for 10 min. 60 μL of the supernatant was placed in an injection vial and injected on the machine for analysis.

Chromatographic conditions, Mass specs conditions and Statistical methods are detailed in the references [45, 46]. For example, the PCA principal component and OPLS-DA partial least squares method are obtained through statistical software (https://www.omicshare.com) in Mataboanalysis 3.0 (https://www.metaboanalyst.ca/) and GENE DENOVO Biological Company cloud platform. Discriminant analysis results. Lipid metabolites meeting P-values ≤0.05 + fold change ≥1.5 or ≤0.667 are defined as significantly different lipids.

### MTT determination

Caco-2 cells were seeded into 96-well culture plates. The cells were then treated with AB23A (0, 10, 100, 1000, or 10000 μM) for 24 h, and then incubated with 0.5 mg/mL MTT for 4 h at 37° C. A multifunctional microplate reader (Biotek Synergy2, USA) was used to measure absorbance at a wavelength of 490 nm. Three independent experiments were performed.

### Real-time quantitative polymerase chain reaction (RT-qPCR)

Total RNA from tissues and cells was extracted using Trizol reagent (Thermo Fisher, USA). The NanoDrop ™ One/One C ultra-micro ultraviolet spectrophotometer (Thermo Fisher, USA) was used to detect the purity and concentration of the extracted total RNA. Then, 1 μg of RNA was converted into cDNA by using HiScript II 1^st^ Strand cDNA Synthesis Kit (Vazyme, China), and the cDNA concentration was detected using NanoDrop ™ One. Reactions were performed on the QuantStudio ™ 6 Flex real-time PCR system using the SYBR ™ GREEN fluorescent dye method. The sequences of the RT-qPCR primers used are as follows: LXRα, forward 5′-TGAGGGAGGAGTGTGTGCTGTC-3′ reverse 5′-TGGCAGGACTTGAGGAGGTGAG-3′; ABCA1, forward 5′- CGTTTCCGGGAAGTGTCCTA-3′ reverse 5′- GCTAGAGATGACAAGGAGGATGGA-3′; ABCG5, forward 5′-CTGAGTCCAGAGGGAGCCAGAG-3′ reverse 5′-CACGGTTGCTGACGCTGTAGG-3′;ABCG8, forward 5′-CCAACTGCTGCCCAACCTGAC-3′ reverse 5′-GCTCGGCGATTACGTCTTCCAC-3′; ACAT2, forward 5′-GCCAGCACACTGAACGATGGAG-3′ reverse 5′-TGGGGTCTACGGCAGCATCAG-3′; SREBP, forward 5′-CTGGCACCGTTGTCTGGATTGG-3′ reverse 5′-TGGGCTCTGTTCCGTCACCTG-3′; HMGCR, forward 5′-GCCGTCATTCCAGCCAAGGTG-3′ reverse 5′-TTTGCTGCGTGGGCGTTGTAG-3′; MTP, forward 5′-AGAAGCTGGCTGGCCTGGTAG-3′ reverse 5′-GCTGCCGCAGTAAGAAGTGGAG-3′; GAPDH, forward 5′-CGGAGTCAACGGATTTGGTCGTAT-3′ reverse 5′-AGCCTTCTCCATGGTGGTGAAGAC-3′. The specificity of all PCR products was evaluated by melting curve analysis. Relative gene expression was analyzed using the 2^-ΔΔCt^ method and normalized with GAPDH as an internal control.

### Western blot analysis

Proximal jejunum tissue and Caco-2 cells were lysed using RIPA lysate and Phenylmethanesulfonyl fluoride (PMSF; Solarbio Life Sciences, Beijing, China) (100: 1). BCA detection kit (CWBIO, Beijing, China) detects protein concentration. Proteins were then separated on a 10% gel (20μg per lane) using sodium lauryl sulfate-polyacrylamide gel electrophoresis (SDS-PAGE, Solarbio Co., Beijing, China) (120V, 90minutes). The relevant proteins were then transferred to a 0.45 μm polyvinylidene fluoride membrane (PVDF, Formex, Darmstadt, Germany). Thereafter, the membrane was blocked in Tris buffered saline solution containing 5% skimmed milk powder and 0.1% Tween-20 (TBS-T) at 20° C for 2 h, and then blocked with anti-LXRα, NPC1L1, ABCG5, ABCG8, ACAT2 and β-actin (1: 1000 dilution) (Abcam, Cambridge, UK) was gently shaken at 4° C overnight. The next day, the membrane was rinsed 3 times with TBS-T (10min each time) and incubated with horseradish peroxidase-conjugated secondary antibody (diluted to 1: 5000, Biosharp, Beijing, China) at room temperature for 2 h. Finally, protein bands were visualized by enhanced chemiluminescence (ECL; Merck Millipore, Darmstadt, Germany) and relative protein levels were quantified using Image Lab software.

### Immunofluorescence

Tissue sections or cells were fixed in 4% paraformaldehyde solution for 30 min, TBS-T was rinsed 3 times (10 min each time), the sections were blocked in 5% BSA for 2 h, and then anti-LXRα, NPC1L1, ABCG5, ABCG8, ABCA1 (1: 100 dilution) (Abcam, Cambridge, UK) Incubate at 20° C for 2h, rinse with TBS-T 3 times (10min each time), and mix with fluorescent secondary antibody (diluted to 1: 100, CWBIO, Beijing, China) at Incubate for 2 h in 20° C, rinse 3 times with TBS-T (10min each time), incubate with DAPI staining solution for 5 min, and rinse 3 times with TBS-T (10 min each time). Cover film. Five independent experiments were performed.

### Small interfering RNA transfection

Specific small interfering RNAs (siRNAs) against ACAT2 (sense, 5′-GCCAGCACACTGAACGATGGAG-3′; antisense, 5′-GACTACGACGGCATCTGGGGT-3′) or FXR (sense, 5′-GUGGUACUCUCCUGGAAUATT-3′; antisense, 5′-UAUUCCAGGAGAGUACCACTT-3′) were synthesized by the Vazyme Company (Nanjing, China). Caco-2 cells (60%-80% confluent monolayer) were seeded in 12-well plates with 1 ml of standard medium. The following day, the cells were transfected with siRNA duplexes (20 nM final concentration) using Lipofectamine™ RNAiMAX reagent (Invitrogen) according to the manufacturer’s instructions. After 72 h, RT-qPCR and western blot analyses were performed to determine transfection efficiency.

### Statistical analysis

All data were collected from at least three independent experiments and shown as Mean ± SEM (standard error of the mean). One-way ANOVA was used to compare the mean values, and then Student Newman-Keuls (SNK) was post-tested by GraphPad Prism 7 software. A P value of less than 0.05 was considered statistically significant.

## Supplementary Material

Supplementary Figures

Biochemical Indicators

IF data

Lipidomics

Lipids level (TC TG HDL LDL)

Oil red data

WB and RT-PCR data
