# Supplementary material for: Alisol B 23-acetate activates ABCG5/G8 in the jejunum via the LXRα/ACAT2 pathway to relieve atherosclerosis in ovariectomized ApoE-/- mice
Source: Aging (Albany NY). 2020 Nov 25;12(24):25744–66. doi: 10.18632/aging.104185 (PMC7803561; doi:10.18632/aging.104185)
Supplement: Supplementary Figures [file aging-12-104185-s001.pdf]

SUPPLEMENTARY FIGURES

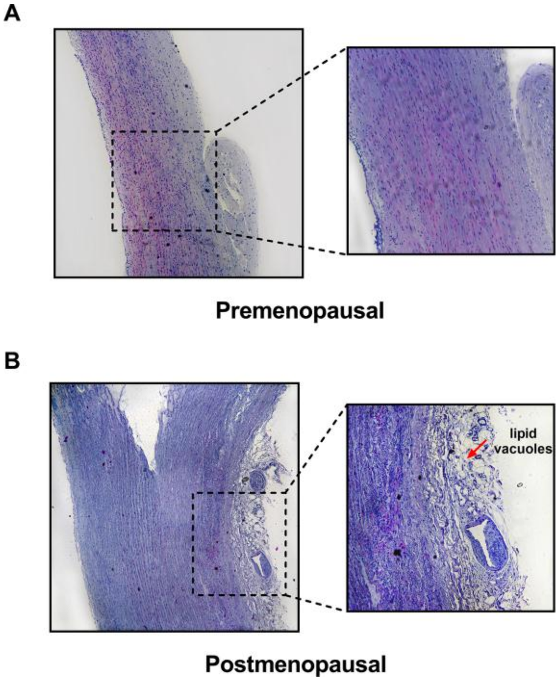

Supplementary Figure 1. Postmenopausal women's ascending aorta lipid deposits increased significantly. (**A**, **B**) Representative histological images of HE staining in ascending aorta in premenopausal women and postmenopausal women. Red arrow, lipid vacuoles. The original magnifications of the images in the same group are 4× (left) and 10× (right).

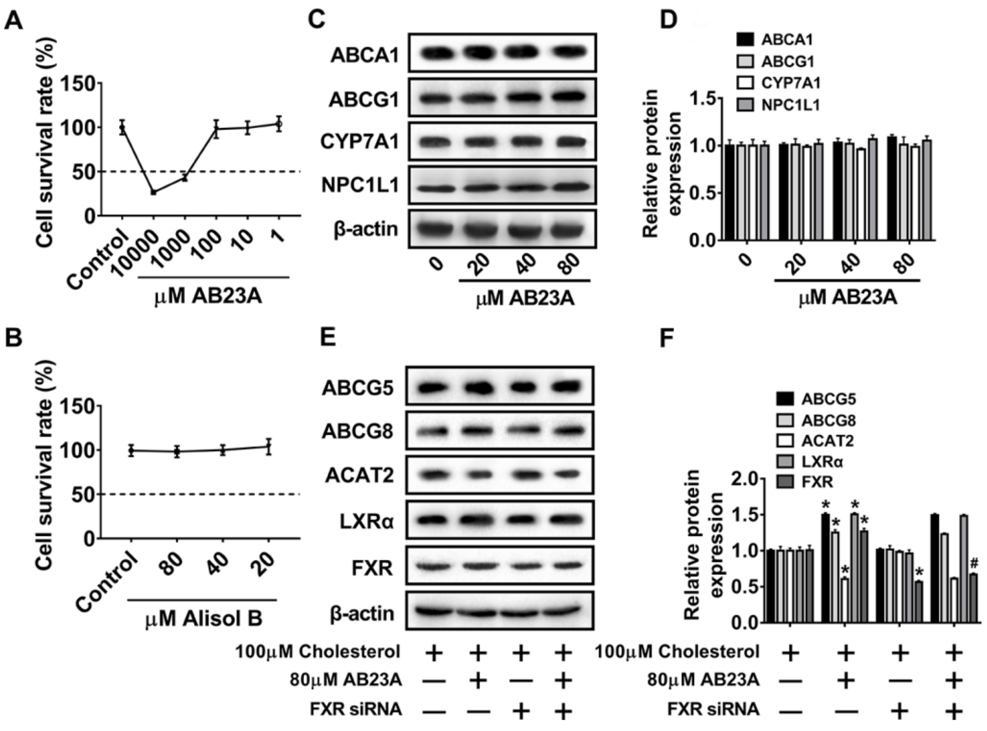

Supplementary Figure 2. Caco-2 cells were used to explore the dosage of AB23A and its effect on the expression of proteins related to lipid metabolism. (**A**) Caco-2 cells were exposed to different concentrations of AB23A (0, 10, 100, 1000, or 10000 μM) for 24 h. The MTT assay was used to detect cell viability. (**B**) Caco-2 cells were exposed to different concentrations of AB23A (0, 20, 40, or 80 μM) for 24 h. Then, the MTT assay was used to detect cell viability. (**C**, **D**) Caco-2 cells cultured under high-fat conditions were exposed to different concentrations of AB23A (0, 20, 40, or 80μM) for 24 h. The protein expressions of ABCA1, ABCG1, CYP7A1 and NPC1L1 in Caco-2 cells from different groups (n=3/group). (**E**, **F**) Caco-2 cells were pretreated with FXR siRNA, Western blot analysis is performed to evaluate the protein levels of ABCG5, ABCG8, ACAT2, LXRα and FXR from different groups (n=3/group). The data are expressed as the mean ± SEM, and the results were obtained from three independent experiments. *P <0.05 compared to the control group; #P <0.05 vs the AB23A only group.

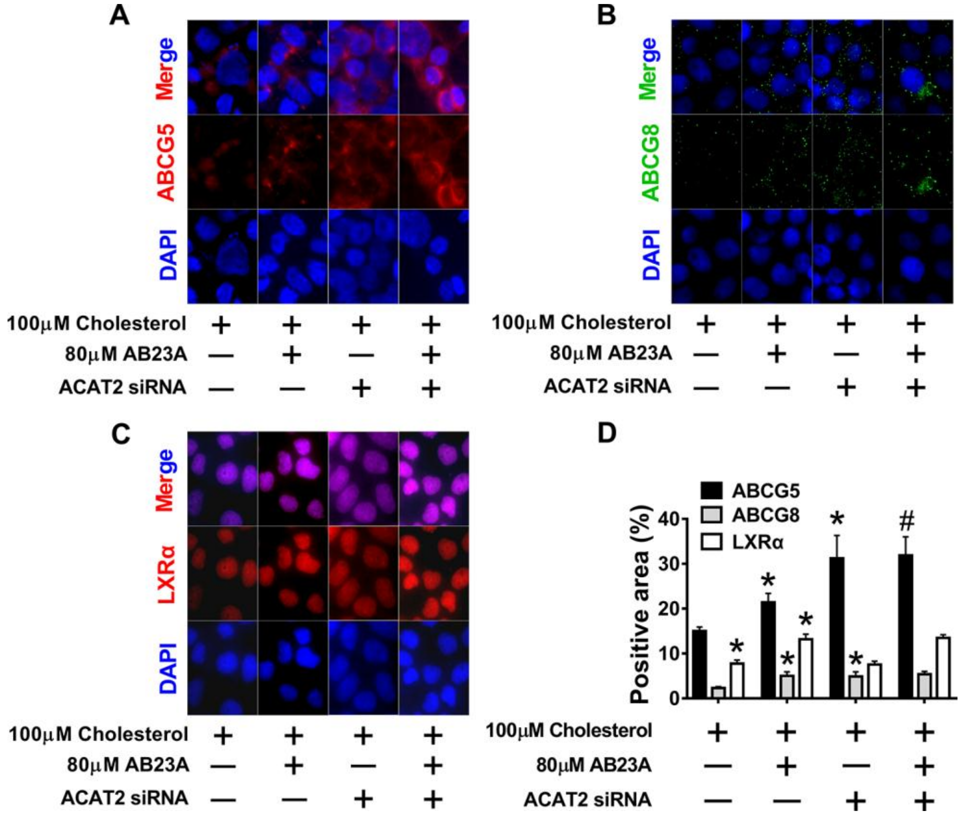

Supplementary Figure 3. The regulation of ABCG5/G8 by AB23A depends on the participation of ACAT2. Caco-2 cells were pre-treated with ACAT2 siRNA, (**A**–**D**) Immunofluorescence were performed to evaluate the protein levels of different groups ABCG5, ABCG8 and LXRα (n=3/group). The data are expressed as the mean ± SEM, and the results were obtained from three independent experiments. *P <0.05 compared to the control group; #P <0.05 vs the AB23A only group.

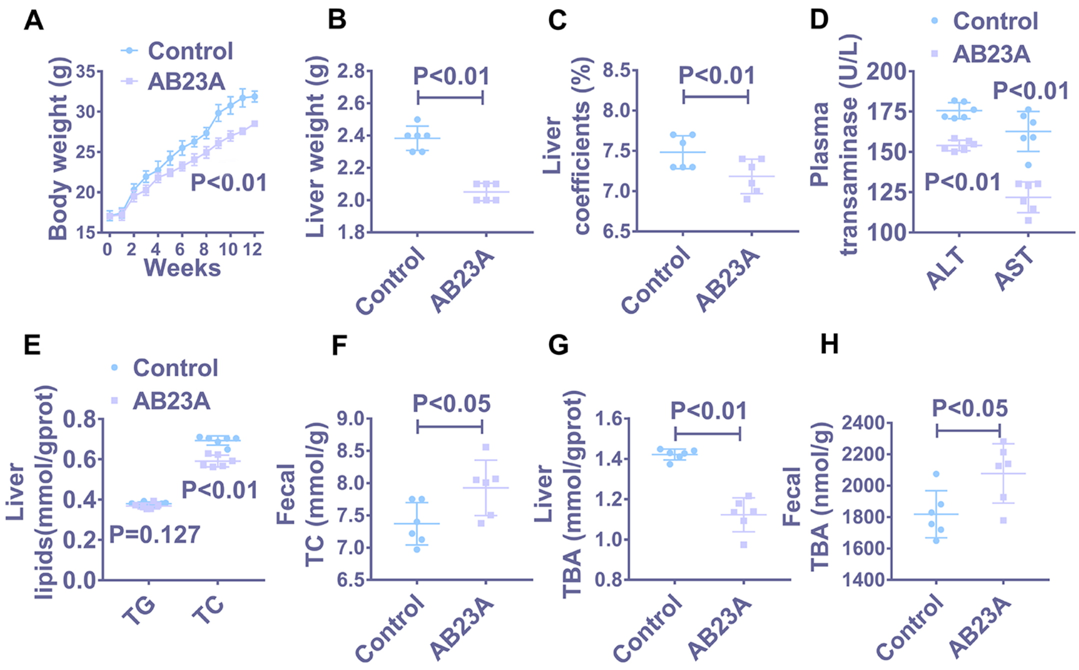

Supplementary Figure 4. AB23A treatment improved the overall parameters of ovariectomized ApoE$^{-/-}$ mice and other biochemical indicators. 8-week-old female ApoE$^{-/-}$ mice were removed from the ovary and given a high-fat diet with saline or AB23A (2.55 mg/kg) daily for 12 weeks. (**A**–**C**) Body weights, liver weights and liver coefficients of mice in different groups. (**D**) Transaminases levels detected in plasma of different groups of mice. (**E**) Kits were used to measure the levels of total triglyceride (TG) and total cholesterol (TC) levels in the livers of mice in different groups. (**F**) Kits were used to measure the levels of total cholesterol (TC) levels in feces of mice in different groups. (**G**, **H**) Kits were used to measure the levels of total bile acids (TBA) in the livers and feces of mice in different groups. The data are expressed as the mean ± SEM, and the results were obtained from three independent experiments. *P <0.05 compared to the control group.

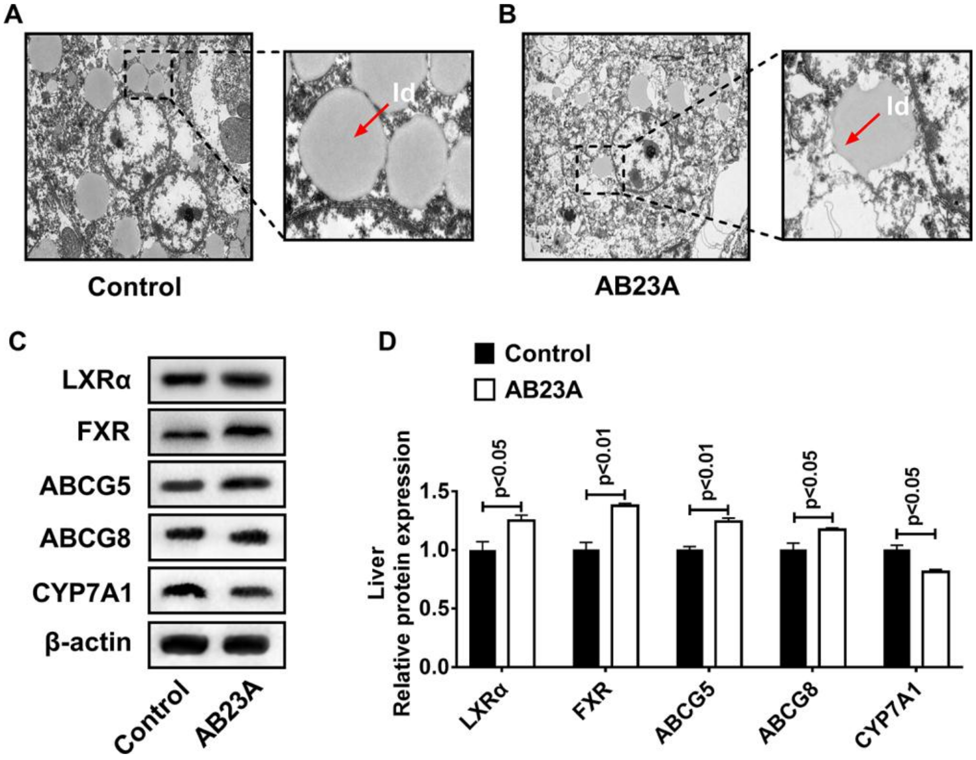

Supplementary Figure 5. AB23A activates FXR in the liver of ovariectomized ApoE$^{-/-}$ mice and reduces lipid accumulation in the liver. 8-week-old female ApoE$^{-/-}$ mice were removed from the ovary and given a high-fat diet with saline or AB23A (2.55 mg/kg) daily for 12 weeks. (**A**, **B**) The TEM image of liver of ovariectomized ApoE$^{-/-}$ mice. Red arrow, lipid droplets. The original magnifications of the images in the same group are 1.2k× (left) and 5.0k× (right). (**C**, **D**) Western blot was used to detect the protein expression of LXRα, FXR, ABCG5, ABCG8 and CYP7A1 in the liver. p-values represent comparisions with controls. The results represent the mean ± SEM (n=3/group).
